# Sleep deficiencies and suicidal ideation across the psychosis continuum

**DOI:** 10.3389/fpsyt.2025.1540497

**Published:** 2025-02-05

**Authors:** Heather M. Wastler, Alexandra M. Blouin, Melissa F. V. Kilicoglu, Melanie Bozzay

**Affiliations:** Department of Psychiatry & Behavioral Health, The Ohio State University Wexner Medical Center, Columbus, OH, United States

**Keywords:** suicidal ideation, sleep, psychosis, clinical high-risk, first-episode, serious mental illness

## Abstract

**Objective:**

Sleep deficiency, a broad term that encompasses sleep disorder symptoms and other aspects of disturbed sleep, is an established risk factor for suicide. Although several studies have examined the relationship between sleep, suicidal ideation, and suicide attempts among individuals with psychotic disorders, few studies have focused on the early stages of illness. The current study addressed this gap in the literature by examining the relationship between sleep deficiencies, recent suicidal ideation, and lifetime suicidal behavior across the psychosis continuum.

**Method:**

A retrospective chart review was used to obtain relevant data for individuals at clinical high-risk for psychosis (CHR-P), individuals with first-episode psychosis (FEP), and individuals with longstanding illness.

**Results:**

Results indicate that sleep deficiencies are prevalent across all stages of illness, though individuals at CHR-P have significantly higher rates of sleep deficiency than individuals with FEP and longstanding psychosis. Additionally, there was a strong relationship between sleep deficiencies and suicidal ideation across the entire sample.

**Conclusions:**

Further research is needed to clarify the specific nature of these sleep deficiencies and to elucidate the mechanisms by which sleep deficiencies might increase risk for suicide in this population.

## Introduction

Psychotic disorders, including schizophrenia spectrum and affective disorders with psychotic features, are considered some of the most devastating health conditions that a person can experience in their lifetime, leading to significant global disease burden (1), impaired functional outcome (2), and even premature death (3). Epidemiological studies have consistently shown high rates of premature mortality among individuals with psychotic disorders, with an estimated 11-13 years of life lost compared to the general population (4). Suicide is a major contributor to these premature deaths, with an estimated worldwide suicide rate of approximately 352 per 100,000 person-years in this population (5). Prevalence rates of suicidal ideation and attempts are even higher, with approximately 27% attempting suicide (6) and 35% thinking about suicide (7) in their lifetime. Importantly, leading models of psychosis suggest that the disease progresses over time, with many researchers recognizing distinct stages of illness (e.g. clinical high-risk for psychosis or the prodromal phase, early psychosis, and longstanding illness) (8, 9). Studies have repeatedly demonstrated that the negative outcomes associated with psychosis cluster in the early stages of illness (10–14), with research showing that suicide risk is greatest during the first few months to years after initial diagnosis (15, 16).There is even growing evidence that this risk might actually precede illness onset. For instance, approximately 6% of individuals with schizophrenia engaged in suicidal behavior during the two years prior to their initial diagnosis (16); similarly, 6-18% of individuals with first-episode (FEP) engaged in deliberate self-harm prior to their diagnosis (15, 17) and approximately 32% had a history of suicidal ideation (15). Consistent with this notion, meta-analyses have found high rates of suicidal thoughts and behaviors among individuals at clinical high-risk for developing a psychotic disorder (CHR-P), with 66% reporting recent suicidal ideation and up to 30% engaging in suicidal behavior in the past six months (18–20). These data indicate that suicide risk is a major concern across the entire psychosis continuum, highlighting the need for research to better understand risk factors that contribute to suicide across all stages of illness (21).

Sleep deficiency, a term that encompasses sleep disorders (i.e., insomnia, hypersomnia, circadian rhythm sleep-wake) and various aspects of disturbed sleep (i.e., insufficient sleep, mistimed sleep, nocturnal wakefulness), is one of many risk factors for suicide in the general population (22–24). Importantly, sleep deficiencies are quite common among individuals with psychosis, presenting in many different ways (e.g., poor subjective sleep quality, greater number of nocturnal wakenings, insomnia, etc.) and presenting across all stages of illness including CHR-P, FEP, and longstanding psychosis (25, 26). Studies generally support a relationship between sleep deficiencies and suicide risk among individuals with FEP (27–29) and longstanding psychosis (30–32). However, surprisingly few studies have focused on sleep deficiency and suicide risk among individuals at CHR-P. The first study in this area involved participants with FEP retrospectively reporting on suicidality and symptoms experienced during their prodromal phase of illness (i.e., when they were CHR-P, before meeting criteria for a FEP) (33). Results indicated that patients with suicidal ideation had greater sleep deficiencies during their prodromal phase compared to those without suicidal ideation (33). Importantly, however, this study was limited by a retrospective design, which required participants to recall sleep experiences that may have occurred up to 6 years prior. Although Andriopoulos and colleagues call for additional research in this area, to our best knowledge there has only been one other study to examine the relationship between sleep and suicide outcomes among individuals at CHR-P. This study demonstrated a relationship between sleep disturbance suicidal ideation, and suicide attempts in CHR-P (34). These studies support the need for more research in this area as sleep deficiencies (25) and suicidality (18, 35, 36) exist across all stage of psychosis and are highly prevalent among individuals at CHR-P (18, 37). The study of sleep deficiencies across the psychosis continuum is an important area for further study, as sleep might be useful to incorporate in suicide risk assessments and because sleep is a modifiable treatment target that could be critical for reducing suicide in this vulnerable population, who typically receives care in specialized treatment settings.

The current study sought to address this gap by examining the relationship between sleep deficiencies, suicidal ideation, and suicide attempts across the psychosis continuum. We capitalize on real-world data collected as part of standard clinical care in a psychosis clinic that treats CHR-P, FEP, and longstanding psychosis, allowing us to examine whether clinician-documented sleep deficiencies can serve as a proxy risk factor for suicide across the psychosis continuum. Based on the existing literature (27, 31, 33), we hypothesized that clinician-documented sleep deficiencies would increase the odds of suicidal ideation and behavior even when controlling for relevant covariates.

## Materials and methods

### Participants and procedures

The current study is a secondary data analysis of a retrospective chart review study that was designed to identify which stage of psychosis has the highest rate of suicidal ideation and behavior. Data was collected as part of standard clinical care for individuals who enrolled in The Ohio State University Early Psychosis Intervention Center (EPICENTER) program from December 2015 to July 2023; EPICENTER has three clinical programs that treat CHR-P, FEP, and longstanding psychosis. This resulted in data for 194 participants across all three programs. Two participants were excluded due to missing sleep data, resulting in a final sample of 50 individuals at CHR-P, 84 individuals with FEP, and 58 individuals with longstanding psychosis (N=192). Eligibility for the CHR-P program includes meeting criteria for clinical high-risk syndrome assessed using the Structured Interview for Psychosis-Risk Syndromes (SIPS) (38) and being ages 12-25; the CHR-P program enrolls individuals with any of the three psychosis-risk syndromes (Attenuated Positive Symptom Syndrome, Brief Intermittent Psychotic Syndrome, and Genetic Risk and Deterioration Syndrome) as well as individuals across any of the four status specifiers (progression, persistence, partial remission, and full remission). Eligibility for the FEP program includes meeting diagnostic criteria for a schizophrenia spectrum (e.g. schizophrenia, schizoaffective, schizophreniform, etc.) or affective disorder with psychotic features (e.g. bipolar disorder with psychotic features, major depression with psychotic features, etc.), experiencing a psychosis onset within the past 5 years, and being ages 15-35. Eligibility for the longstanding psychosis program includes meeting diagnostic criteria for a schizophrenia spectrum or affective disorder with psychotic features and experiencing a psychosis onset greater than 5 years ago. Exclusion criterion for all three programs includes any evidence of a primary intellectual disorder. The EPICENTER intake includes a set of standard questions that are delivered to all patients. For the current study, we obtained demographics, suicidal ideation, depressed mood, clinician-documented sleep deficiency, and lifetime suicidal behavior from the intake note. This study was approved by The Ohio State University Institutional Review Board; requirement for consent was waived by the ethics committee.

### Measures

Suicide outcomes were assessed using the Patient Safety Screener (PSS-3), a brief clinician administered measure that assesses depressed mood, suicidal ideation, and lifetime suicide attempt(s) (39, 40). When a lifetime attempt is endorsed, a follow up question is administered to inquire about when the attempt occurred. For the current study, we coded binary responses for each item to indicate the presence of past two-week depressed mood, past two-week suicidal ideation, and lifetime suicidal behavior. If an attempt was documented, additional details about the behavior (i.e., what happened and when it occurred) were obtained from the open-text sections of the intake note to confirm the presence of suicidal behavior. Of note, aborted and interrupted attempts were included as lifetime suicidal behavior because some clinicians did not document sufficient detail for us to determine whether the patient stopped before or after taking the action. Thus, we use the term suicidal behavior, rather than attempts, to more accurately represent this broader range of behavior.

Clinician-documented sleep deficiencies were obtained from the mental status section of the intake note. This multiple-select item allows providers to document any of the following sleep indicators reported by their patients: decreased more than normal, does not feel rested, fitful, good, improved, increased more than normal, no change, poor with difficulty initiating and maintaining sleep, poor with difficulty initiating sleep, poor with difficulty maintaining sleep, and/or wants to sleep all the time. Many providers also included additional open-text details about patient sleep. Thus, raw text was obtained from this section of the note and responses were coded to indicate the presence or absence of clinician-documented sleep deficiencies. Specifically, a binary variable was created to indicate the presence/absence of any sleep-related deficiency (e.g. difficultly falling asleep, difficulty maintaining sleep, increased sleep, etc.). For descriptive purposes, the authorship team also coded factors that could indicate the presence or absence of sleep disorder symptoms (possible insomnia or hypersomnia symptoms), changes in sleep patterns (increased or decreased), and/or other factors (e.g., nightmares, phase-shift, etc.).

### Statistical analyses

Chi-Square tests were used to examine whether clinician-documented sleep deficiencies varied across the psychosis continuum. Separate Chi-Square tests were conducted to compare CHR-P to FEP, CHR-P to longstanding psychosis, and FEP to longstanding psychosis. Next, we used logistic regression to examine the relationship between sleep deficiency and suicide outcomes collapsed across the entire sample. Logistic regression models accounted for age, sex assigned at birth, and depressed mood; these covariates were selected because 1) there are age-related eligibility criteria for some EPICENTER programs, 2) there are well-established sex differences in suicidal thoughts and behaviors in the general population (41), and 3) depression is a risk factor for suicidal thoughts and behaviors in psychosis (13, 42). Two separate models were used to examine recent suicidal ideation and lifetime suicidal behavior as outcomes. Analyses were conducted using SPSS Version 29.0.1.0.

## Results

Demographics are summarized in **Table 1**. A total of 37 (19.3%) participants endorsed past two-week suicidal ideation and 45 (23.4%) endorsed lifetime suicidal behavior. The sample was predominantly Male (57.8%), White (65.5%), and Non-Hispanic or Latino (93.2%). The three groups did not differ in terms of sex assigned at birth (χ^2^ (2) =5.73, *p*=0.06), race (χ^2^ (12) =4.67, *p*=0.97), or ethnicity (χ^2^ (6) =7.78, *p*=0.26). The three groups differed in age (*F*(2, 189)=100.04, *p*<0.001), with the longstanding psychosis group being significantly older than the FEP (*p*<0.001) and CHR-P (*p*<0.001) groups; the FEP and CHR-P groups did not differ in age (*p*=0.137).

**Table 1:** Participant characteristics.

Variable	Tota (n=192)	CHR-P (n=50)	FEP (n=84)	Longstanding (n=58)
Age (M ± SD)	25.72 ± 10.27	19.32 ± 2.47	21.90 ± 4.34	36.78 ± 11.79
Sex assigned at birth				
Male	111 (57.8%)	23 (46.0%)	56 (66.7%)	32 (55.2%)
Female	81 (42.2%)	27 (54.0%)	28 (33.3%)	26 (44.8%)
Race				
African American or Black	33 (17.2%)	7 (14.0%)	16 (19.0%)	10 (17.2%)
Asian	12 (6.3%)	3 (6.0%)	6 (7.1%)	3 (5.2%)
Caucasian or White	126 (65.6%)	36 (72.0%)	54 (64.3%)	36 (62.1%)
More than one race	10 (5.2%)	2 (4.0%)	4 (4.8%)	4 (6.9%)
Other	4 (2.1%)	1 (2.0%)	2 (2.4%)	1 (1.7%)
Unknown to patient	5 (2.6%)	1 (2.0%)	1 (1.2%)	3 (5.2%)
Refused to answer	2 (1.0%)	0 (0.0%)	1 (1.2%)	1 (1.7%)
Ethnicity				
Not Hispanic or Latino	179 (93.2%)	48 (96.0%)	81 (96.4%)	50 (86.2%)
Hispanic or Latino	4 (2.1%)	1 (2.0%)	1 (1.2%)	2 (3.4%)
Unknown to patient	7 (3.6%)	1 (2.0%)	1 (1.2%)	5 (8.6%)
Refused to answer	2 (1.0%)	0 (0.0%)	1 (1.2%)	1 (1.7%)
Depressed Mood	91 (47.4%)	36 (72.0%)	38 (45.2%)	17 (29.3%)
Suicidal Ideation	37 (19.3%)	17 (34.0%)	14 (16.7%)	6 (10.3%)
Lifetime Suicidal Behavior	45 (23.4%)	9 (18.0%)	20 (23.8%)	16 (27.6%)
Sleep Deficiencies				
Sleep Disorder Symptoms	33 (17.2%)	14 (28.0%)	9 (10.7%)	10 (17.2%)
Sleep Pattern Changes	16 (8.3%)	13 (26.0%)	2 (2.4%)	1 (1.7%)
Other Factors	25 (13.0%)	10 (20.0%)	11 (13.1%)	4 (6.9%)

The “other sleep factors” category was used for participants who reported sleep deficiencies, but the nature of their sleep deficiencies either 1) could not be classified as sleep disorder/sleep pattern symptoms or 2) there was insufficient information to categorize them in more detail. Examples include the following: not feeling well rested, fitful sleep, sleep difficulties (without details), fragmented sleep, etc. Participants could be classified as having more than one type of sleep deficiency, with 16 participants in the total sample being categorized as having two types of sleep deficiencies; no participant had all three types of sleep deficiencies documented in their chart.

Results from the Chi-Square analyses indicated that the three groups differed as a function of clinician-documented sleep deficiencies (χ^2^ (2) = 18.53, *p*<0.001, *V* = 0.31). *Post-hoc* Chi-Square tests revealed that the CHR-P group was more likely to have documented sleep deficiencies than the FEP group (χ^2^(1) = 12.55, *p <*0.001, *V* = 0.31) and the longstanding psychosis group, (χ^2^(1) = 14.45, *p <*0.001, *V* = 0.37). Clinician-documented sleep deficiencies did not significantly differ across FEP and longstanding psychosis groups (χ^2^ (1) = 0.47, *p*=0.49, *V* = 0.06). More specifically, 54.0% of the CHR-P group had sleep deficiencies, whereas 23.8% of the FEP and 19.0% of the longstanding psychosis group reported sleep deficiencies. Overall, possible sleep disorder symptoms were commonly documented across the entire sample (17.2%). Changes in sleep patterns were less common (8.3%). Other sleep factors occurred in approximately 13.0% of the total sample. The CHR-P group had the highest prevalence of all three types of sleep deficiency concerns.

**Table 2** summarizes results from the logistic regression examining the relationship between suicidal ideation and clinician-documented sleep deficiencies collapsed across the entire sample. The full model including all predictors was significant (χ^2^ (4) = 56.03, *p <*0.001), correctly identifying 83.3% of cases and accounting for 25.3% (Cox & Snell R^2^) to 40.5% (Nagelkerke R^2^) of the variance in suicidal ideation. Suicidal ideation was associated with sleep deficiencies, even when including demographics and depressed mood in the model, (OR= 3.10, 95% CI [1.33, 7.23]). Of note, very wide confidence intervals were observed for depressed mood, likely because 94.6% of participants with suicidal ideation also endorsed a depressed mood; only two participants with ideation denied a depressed mood (5.4%).

**Table 2:** Suicidal ideation and clinician-documented sleep deficiencies.

	B	SE	p	OR	95% CI	
					LL	UL
Age	-0.03	0.03	0.257	0.97	0.93	1.02
Sex	-0.20	0.44	0.647	0.82	0.35	1.93
Depressed Mood	3.19	0.76	<0.001	24.34	5.49	107.83
Sleep Deficiencies	1.13	0.43	0.009	3.10	1.33	7.23

A model with group included as a predictor indicated that group did not significantly predict ideation (*p*=0.749). Similarly, the interaction of sleep deficiencies and group was non-significant (*p*=0.977).

**Table 3** summarizes results from the logistic regression model examining the relationship between clinician-documented sleep deficiency and lifetime suicidal behavior collapsed across the entire sample. The full model including all predictors was not significant (χ^2^(4) = 8.07, *p*=0.089). Only age was associated with lifetime suicidal behavior. Sleep deficiency and recent depressed mood were not associated with lifetime suicidal behavior.

**Table 3:** Lifetime suicidal behavior and clinician-documented sleep deficiencies.

	B	SE	p	OR	95% CI	
					LL	UL
Age	0.04	0.02	0.022	1.04	1.01	1.07
Sex	0.33	0.36	0.351	1.40	0.69	2.81
Depressed Mood	0.49	0.38	0.194	1.63	0.78	3.42
Sleep Deficiencies	0.07	0.39	0.851	1.08	0.50	2.31

A model with group included as a predictor indicated that group did not significantly predict suicidal behavior (*p*=0.489). Similarly a model examining the interaction of sleep deficiencies and group was non-significant (*p*=0.339).

## Discussion

This retrospective chart review examined the relationship between clinician-documented sleep deficiencies, recent suicidal ideation, and lifetime suicidal behavior among individuals at CHR-P, individuals with FEP, and individuals with longstanding psychosis. Results support a strong relationship between clinician-documented sleep deficiency and suicidal ideation across the psychosis continuum, with sleep deficiencies increasing the odds of suicidal ideation by three-fold, even when including demographics and depressed mood in the model. Although this study represents an analysis of clinician-documented sleep deficiencies in the absence of formal measures of sleep symptoms, these findings are consistent with the broader literature demonstrating a relationship between sleep deficiency and suicidal ideation in longstanding psychosis (31, 32) and FEP (27–29). Our findings expand this literature by also including individuals at CHR-P, further supporting a relationship between sleep deficiencies and suicidal ideation across the psychosis continuum.

Surprisingly, we found that clinician-documented sleep deficiencies were not associated with lifetime suicidal behavior. These results are in contrast to prior research in the general population, which has found that different facets of sleep deficiency are related to increased risk for suicidal behavior (31, 43). Our findings suggest that sleep deficiencies are associated with past two week ideation, but not lifetime suicidal behavior among individuals with psychosis. Nonetheless, it is possible that we did not identify associations between sleep deficiency and lifetime suicidal behavior because we used measures of recent clinician-documented sleep deficiency, but lifetime suicidal behavior. It is still possible that sleep deficiencies might have a proximal relationship with suicidal behavior even if individuals with lifetime attempts do not exhibit higher rates of sleep deficiencies in our sample. Future intensive longitudinal research (e.g., ecological momentary assessment paired with actigraphy) that examines how sleep impacts suicide risk the following day is needed to determine whether sleep deficiencies have a proximal relationship with suicidal behavior among individuals with and at risk for psychosis.

The current study also compared the frequency of clinician-documented sleep deficiencies across stages of illness. Results indicate that individuals at CHR-P have the highest rates of sleep deficiencies, with over half of participants having some level of sleep deficiency documented in their chart during the intake visit. To our best knowledge, only one other study has compared sleep deficiencies across stages of illness (25). In contrast to the current study, this meta-analysis found no group differences across stages of illness (25). Importantly, the current study used a proxy measure of sleep deficiency, which could index a number of other concerns in addition to potentially indexing more severe sleep disturbance, as measured by Bagautdinova and colleagues (2023). For example, higher rates of sleep deficiencies in the current study could index medication side effects, comorbidities, life stress, or even patient’s tendencies to discuss sleep-related concerns with their providers. Thus, it is possible that our broad definition of sleep deficiencies captured some other sleep-related concern that is even more pronounced among individuals at CHR-P. Additional research is needed to determine whether individuals at CHR-P do in fact have higher rates of sleep deficiencies than other stages of illness and if so, why that might be the case. It is also important to note that although our CHR-P group had the highest rates of sleep deficiencies, the current study did find rates of approximately 24% in the FEP group and 19% in the longstanding psychosis group. Thus, even with the CHR-P group having the highest prevalence in this study, we did find that sleep deficiencies are common across all stages of illness. Taken together with our finding that sleep deficiency is associated with suicidal ideation across the entire sample, this study highlights the potential importance of sleep deficiencies across all stages of illness. Further research with direct sleep measures is needed to disentangle whether there are some sleep deficiencies that occur across all stages of illness versus others that might be stage-specific. Such work could also clarify whether our findings represent true sleep disturbance or if they are better accounted for by other related concerns (e.g., medication side effects, comorbidities, stress).

Results of the current study should be interpreted within the context of several limitations. As noted above, we were limited by the use of a proxy sleep measure, relying on clinician-reported data from the mental status section of the intake note. Additional research with validated self-report measures, clinical interviews, and/or objective sleep indices (e.g., actigraphy, polysomnography) are critical for validating our findings and clarifying the nature of sleep deficiency as it relates to suicide risk in psychosis. Relatedly, the mental status section of the intake note did not specify the assessment timeframe for sleep. Given the nature of mental status exams, it is likely that clinicians assessed recent sleep deficiencies. Nonetheless, we are unable to verify the exact assessment timeframe, which limits our ability to determine whether the sleep deficiencies occurred around the same time as suicidal ideation, which was assessed for the past two weeks. Similarly, review of the raw data on suicidal behavior indicated substantial variability in the way clinicians documented and defined suicide attempts. Thus, it is possible that some aspects of suicidal behavior (e.g., aborted and interrupted attempts) were under-reported in our data. Additional research using more comprehensive suicide measures is needed to further examine the relationship between sleep deficiency and suicide attempts across the psychosis continuum. Next, due to our retrospective chart review design, we were limited by the types and amount of data we were able to collect. Specifically, we were unable to obtain data on other potential variables that may influence the relationship between sleep and suicide risk. Sleep deficiencies are known to influence a number of outcomes for individuals with psychosis (e.g. positive symptoms, functioning, stress, cognition) (26, 37, 44, 45). Thus, it is possible that sleep affects suicide risk through an indirect pathway by influencing these other variables. For instance, poor sleep might lead to heightened stress reactivity, which might lead to suicidal ideation. Additional research is needed to delineate potential mechanisms for the relationship between sleep and suicidal ideation in psychosis. Relatedly, the current study did not obtain data about psychotropic medication use. Additional research is needed to examine whether psychotropic medications influence the relationship between sleep deficiencies and suicide risk in psychosis. Lastly, the current study was cross-sectional, preventing us from making inferences about causality and directionality. Additional longitudinal research that uses fine-grained assessment tools (e.g., ecological momentary assessment, actigraphy) is needed to examine whether there is a bidirectional relationship between sleep and suicidal ideation in psychosis. These fine-grained tools would also be useful for determining whether sleep deficiencies lead to proximal changes in suicide risk across the psychosis spectrum.

Notwithstanding these limitations, the current study suggests that sleep deficiencies are commonly documented in standard clinical care settings for psychosis. Importantly, clinician-documented sleep deficiencies were associated with an increased likelihood of suicidal ideation, even when accounting for depressed mood. Additional research is needed to clarify the specific nature of these sleep deficiencies and to elucidate the mechanisms by which sleep deficiency might increase risk for suicide among individuals across the psychosis continuum.

## Data Availability

The datasets presented in this article are not readily available because a waiver of consent was obtained and participants did not provide permission for their data to be shared. Requests to access the datasets should be directed to heather.wastler@osumc.edu.
